# Comparative genomic analysis reveals occurrence of genetic recombination in virulent *Cryptosporidium hominis* subtypes and telomeric gene duplications in *Cryptosporidium parvum*

**DOI:** 10.1186/s12864-015-1517-1

**Published:** 2015-04-18

**Authors:** Yaqiong Guo, Kevin Tang, Lori A Rowe, Na Li, Dawn M Roellig, Kristine Knipe, Michael Frace, Chunfu Yang, Yaoyu Feng, Lihua Xiao

**Affiliations:** State Key Laboratory of Bioreactor Engineering, School of Resources and Environmental Engineering, East China University of Science and Technology, Shanghai, 200237 China; Division of Foodborne, Waterborne, and Environmental Diseases, Centers for Disease Control and Prevention, Atlanta, GA 30333 USA; Division of Scientific Resources, Centers for Disease Control and Prevention, Atlanta, GA 30333 USA; Division of Global HIV/AIDS, Centers for Disease Control and Prevention, Atlanta, GA 30333 USA

**Keywords:** *Cryptosporidium*, Genomics, Whole genome sequencing, Genetic recombination, Virulence

## Abstract

**Background:**

*Cryptosporidium hominis* is a dominant species for human cryptosporidiosis. Within the species, IbA10G2 is the most virulent subtype responsible for all *C. hominis*–associated outbreaks in Europe and Australia, and is a dominant outbreak subtype in the United States. In recent yearsIaA28R4 is becoming a major new subtype in the United States. In this study, we sequenced the genomes of two field specimens from each of the two subtypes and conducted a comparative genomic analysis of the obtained sequences with those from the only fully sequenced *Cryptosporidium parvum* genome.

**Results:**

Altogether, 8.59-9.05 Mb of *Cryptosporidium* sequences in 45–767 assembled contigs were obtained from the four specimens, representing 94.36-99.47% coverage of the expected genome. These genomes had complete synteny in gene organization and 96.86-97.0% and 99.72-99.83% nucleotide sequence similarities to the published genomes of *C. parvum* and *C. hominis*, respectively. Several major insertions and deletions were seen between *C. hominis* and *C. parvum* genomes, involving mostly members of multicopy gene families near telomeres. The four *C. hominis* genomes were highly similar to each other and divergent from the reference IaA25R3 genome in some highly polymorphic regions. Major sequence differences among the four specimens sequenced in this study were in the 5′ and 3′ ends of chromosome 6 and the gp60 region, largely the result of genetic recombination.

**Conclusions:**

The sequence similarity among specimens of the two dominant outbreak subtypes and genetic recombination in chromosome 6, especially around the putative virulence determinant gp60 region, suggest that genetic recombination plays a potential role in the emergence of hyper-transmissible *C. hominis* subtypes. The high sequence conservation between *C. parvum* and *C. hominis* genomes and significant differences in copy numbers of MEDLE family secreted proteins and insulinase-like proteases indicate that telomeric gene duplications could potentially contribute to host expansion in *C. parvum*.

**Electronic supplementary material:**

The online version of this article (doi:10.1186/s12864-015-1517-1) contains supplementary material, which is available to authorized users.

## Background

*Cryptosporidium* spp. inhabit the brush borders of the gastrointestinal and respiratory epithelium of various vertebrates, causing enterocolitis, diarrhea, and cholangiopathy in humans [1]. Immunocompetent children and adults with cryptosporidiosis usually have a short-term illness accompanied by watery diarrhea, nausea, vomiting, and weight loss. In immunocompromised persons, however, the infection can be protracted and life-threatening [2]. Cryptosporidiosis is one of the most important causes of moderate-to-severe diarrhea and diarrhea-associated deaths in children in developing countries [3] and a major cause for waterborne and foodborne outbreaks of human illness in industrialized nations [4,5]. In the United States the number of reported cases of cryptosporidiosis has increased more than twofold since 2005 [6-9]. Currently, it is estimated that there are approximately 750,000 annual cases of cryptosporidiosis in the United States [5].

Among the many established *Cryptosporidium* species and genotypes, *C. hominis* and *C. parvum* are the two responsible for greater than 90% of the human cryptosporidiosis cases in most countries. *C. hominis* is largely human-specific and responsible for anthroponotic transmission of cryptosporidiosis. *C. parvum* infects both humans and some farm animals, especially pre-weaned calves and lambs and thus can be transmitted both anthroponotically and zoonotically [10]. Within *C. hominis*, subtype IbA10G2 is the dominant strain for *C. hominis*-associated waterborne outbreaks of cryptosporidiosis in the United States, Europe, and Australia [10-16]. The dominant subtype associated with waterborne cryptosporidiosis outbreaks in the United States since 2005 is a new subtype, IaA28R4 [17-20].

Whole genome sequencing of *Cryptosporidium* spp. has greatly facilitated the development of genotyping, subtyping and multilocus sequence typing (MLST) tools for characterizing the transmission of *C. hominis* and *C. parvum* [21,22]. These tools have played a major role in improving our understanding of cryptosporidiosis epidemiology [10,23]. Nevertheless, genomic studies of *Cryptosporidium* spp. lag far behind those on other related apicomplexan parasites largely because of the lack of effective cultivation and animal models. Thus far, only the genomes of one laboratory isolate each of *C. parvum*, *C. hominis*, and *C. muris* have been sequenced using traditional Sanger sequencing technology [22,24,25]. More recently, the genome of an anthroponotic II subtype (IIcA5G3b) of *C. parvum* serially propagated in immunosuppressed mice has been sequenced using Illumina technology [26]. The lack of whole genome sequence data, especially from field specimens obtained from outbreaks, has hampered our understanding of genetic determinants for host specificity, virulence, and the biological fitness of various *Cryptosporidium* species and *C. parvum* and *C. hominis* subtypes.

In this study, we sequenced the genomes of two dominant outbreak subtypes (IbA10G2 and IaA28R4) of *C. hominis* by using 454 and Illumina technologies. Prior to sequencing, oocysts were isolated directly from field specimens without propagation in laboratory animals, and extracted DNA was amplified to generate enough material for sequencing. Results of this study have (1) filled some gaps in our understanding of *Cryptosporidium* genomics, (2) identified some major deletions and one large insertion in the *C. hominis* genome, and (3) showed the high genetic similarity of the two outbreak subtypes. We have also demonstrated the occurrence of genetic recombination in chromosome 6.

## Results

### *Cryptosporidium hominis* sequence data and *de novo* assemblies

After sequencing using 454 technology, 1,048,412 reads (382.5 Mb) were obtained from specimen 30974 (IbA10G2) and 1,157,140 reads (431.7 Mb) were obtained from specimen 33537 (IaA28R4). They produced an assembly of 8,841,752 bp in 443 contigs for specimen 30974 (N50 = 78,110 bp) and an assembly of 14,065,231 bp in 1,464 contigs for specimen 33537 (N50 = 27,749 bp). Using Illumina paired-end sequencing, 64,449,544 reads (5,780.0 Mb) were obtained from specimen 30976 (IaA28R4) and 30,886,077 reads (2,798.3 Mb) were obtained from specimen 37999 (IbA10G2). They produced an assembly of 22,133,082 bp in 6,140 contigs for specimen 30976 (N50 = 145,968 bp) and 9,054,010 bp in 78 contigs for specimen 37999 (N50 = 406,678 bp; Table 1).

**Table 1:** **Summary of sequence data from whole genome sequencing of four** ***Cryptosporidium hominis*** **specimens in comparison with data from the published** ***C. hominis*** **(TU502) and** ***C. parvum*** **(IOWA) genomes**

**Specimen (gp60 subtype)**	**Technique**	**Total nucleotides**	**Total sequence reads**	**Assembly**						**Average coverage (fold)**
				**# of Contigs**	**Length (bp)**	**Mean (bp)**	**Minimum (bp)**	**Maximum (bp)**	**N50 (bp)**	
30976 (IaA28R4)	Illumina Genome Analyzer IIx 100 bp paired end	5,780,028,818	64,449,544	6,140	22,133,082	3,605	502	1,279,890	145,968	257
37999 (IbA10G2)	Illumina Genome Analyzer IIx 100 bp paired end	2,798,259,889	30,886,077	78	9,054,010	116,077	510	1,029,232	406,678	307
33537 (IaA28R4)	454 GS-FLX Titanium	431,742,212	1,157,140	1,464	14,065,231	9,607	501	154,507	27,749	31
30974 (IbA10G2)	454 GS-FLX Titanium	382,520,957	1,048,412	443	8,841,752	19,959	513	325,032	78,110	43
*C. hominis* TU502 (IaA25R3)	Sanger	-	-	1,422	8,743,570	6,149	251	90,444	14,504	12
*C. parvum* IOWA (IIaA15G2R1)	Sanger	-	-	18	9,102,324	504,874	17,388	1,278,458	1,014,526	13

### Genome coverage and bacterial contamination

**Figure 1:**
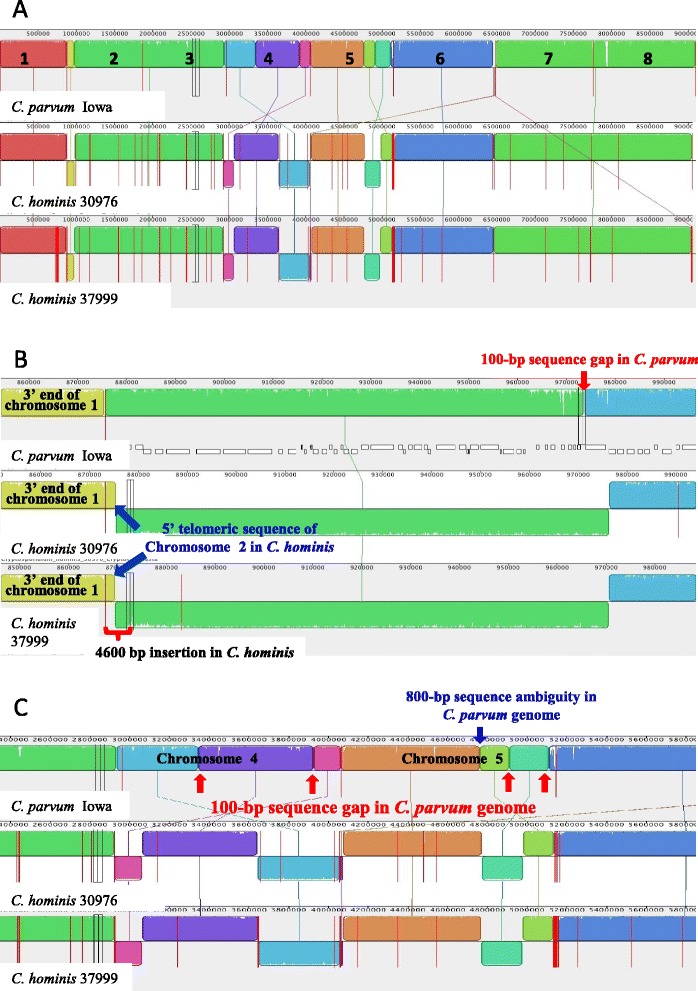
Structural organization of two Illumina-sequenced genomes of *Cryptosporidium hominis* comparing to eight chromosomes (numbered and separated by vertical red lines) of published *Cryptosporidium parvum* genome. The color blocks (known as Locally Collinear Blocks) are conserved segments of sequences internally free from genome rearrangements, whereas the inverted white peaks within each block are sequence divergence between the reference *C. parvum* (IOWA) genome and *C. hominis* genome under analysis. **A**. Coverage of two *C. hominis* genomes showing possible sequence rearrangements in chromosomes 2, 4, 5 and 6. Assembled contigs are bordered by vertical red lines. For specimens 30976, only *Cryptosporidium* contigs were used in mapping. **B**. Possible sequence rearrangements at the 5′ end of chromosome 2. **C**. Possible sequence rearrangements in chromosomes 4 and 5.

Among the two specimens sequenced by 454 technology, 424 of the 443 contigs generated from the IbA10G2 specimen 30974 and 767 of the 1,464 contigs from the IaA28R4 specimen 33537 mapped to the eight chromosomes of the *C. parvum* IOWA isolate, representing 8,816,174 and 8,590,919 bp, thus giving a 96.9% and 94.4% coverage of the genomes, respectively (Table 2, Additional file 1: Figure S1). The *C. parvum* genome was used as the reference because it is the most complete genome fully assembled into eight chromosomes with the aid of a physical HAPPY map generated prior to the sequencing effort [27], and has ~97% sequence similarity to the reference *C. hominis* TU502 (IaA25R3) genome. The latter has 1,422 scaffolds and contigs, but was estimated to have synteny to many contigs in the *C. parvum* IOWA (IIaA15G2R1) genome sequences [25]. For the two specimens sequenced by paired-end Illumina, 64 of the 78 contigs generated from the IbA10G2 specimen 37999 and 45 of the 6,140 contigs from the IaA28R4 specimen 30976 mapped to the eight chromosomes of the *C. parvum* reference genome. The mapped contigs represented 9,041,990 and 9,054,312 bp and thus had 99.34% and 99.47% coverages of the genomes, respectively. In contrast, 1,269 of 1,422 contigs from the published genome of TU502 (IaA25R3) mapped to the eight *C. parvum* chromosomes, representing a 95.5% coverage (Table 2, Figure 1A). No physical map is available for any of the *C. hominis* specimens sequenced to date to aid the assembly of genomic sequences.

**Table 2:** **Coverage of four** ***Cryptosporidium hominis*** **genomes sequenced in this study and sequence similarities to published** ***C. parvum*** **(IOWA) and** ***C. hominis*** **(TU502) genomes**

**Chromosome**	***C. parvum*** **length (bp)**	**37999 (IbA10G2)**					**30976 (IaA28R4)**					**30974 (IbA10G2)**					**33537 (IaA28R4)**					**TU502 (IaA25R3)**			
		**Contigs mapped**	**Length (bp)**	**Coverage (%)**	**Similarity to IOWA (%)**	**Similarity to TU502 (%)**	**Contigs mapped**	**Length (bp)**	**Coverage (%)**	**Similarity to IOWA (%)**	**Similarity to TU502 (%)**	**Contigs mapped**	**Length (bp)**	**Coverage (%)**	**Similarity to IOWA (%)**	**Similarity to TU502 (%)**	**Contigs mapped**	**Length (bp)**	**Coverage (%)**	**Similarity to IOWA (%)**	**Similarity to TU502 (%)**	**Contigs mapped**	**Length (bp)**	**Coverage (%)**	**Similarity to IOWA (%)**
1	875659	15	867575	99.08	96.8	99.79	1	873289	99.73	96.81	99.86	36	863586	98.62	96.84	99.82	54	840604	96	96.93	99.86	124	859754	98.18	96.88
2	985969	7	987017	100.11	96.79	99.58	8	983830	99.78	96.82	99.81	41	970191	98.4	96.81	99.77	69	944487	95.8	96.9	99.82	115	946071	95.95	96.73
3	1099352	13	1098355	99.91	96.89	99.62	13	1096430	99.73	96.86	99.79	40	1081251	98.35	96.86	99.75	87	1064015	96.78	96.98	99.81	158	1079381	98.18	96.78
4	1104417	3	1103687	99.93	96.76	99.72	4	1105075	100.06	96.76	99.8	86	1056974	95.7	96.81	99.74	123	1024384	92.75	96.93	99.82	195	1007110	91.19	96.77
5	1080900	13	1092751	101.1	96.78	99.74	11	1107822	102.49	96.78	99.84	71	1013283	93.74	96.93	99.77	118	940540	87.01	97.09	99.83	186	972978	90.02	96.79
6	1332857	5	1304591	97.88	96.91	99.76	2	1298888	97.45	96.93	99.86	66	1263267	94.78	97.01	99.76	124	1237394	92.84	97.09	99.83	192	1240122	93.04	96.82
7	1278458	5	1268482	99.22	97.19	99.79	4	1269257	99.28	97.2	99.87	27	1267106	99.11	97.18	99.82	79	1258429	98.43	97.25	99.88	124	1282777	100.34	97.18
8	1344712	3	1319172	98.1	96.78	99.76	2	1319721	98.14	96.8	99.83	57	1300516	96.71	96.81	99.78	113	1281066	95.26	96.9	99.81	175	1304075	96.98	96.74
Total	9102324	64	9041990	99.34	96.86	99.72	45	9054312	99.47	96.87	99.83	424	8816174	96.93	96.9	99.78	767	8590919	94.36	97	99.83	1269	8692268	95.49	96.84

Most of the 14 unmapped contigs from specimen 37999 were small (≤1,824 bp) and were sequences of multicopy genes (ex. rRNA units) and genes with paralogs in the genome or large repetitive sequences (ex. fatty acid synthase and cgd5_1210 and cgd5_1220). However, sequences of four contigs (45, 66, 74, and 77) had no similarity to any published sequences, and one contig (#76) had 95% sequence similarity to a 500-bp region of the genome of *Strentrophomonas maltophilia* (CP002986). Similarly, most of the unmapped contigs from specimen 30974 were small (≤3,170 bp) and were sequences of multicopy genes (ex. rRNA units), genes with paralogs in the genome and large repetitive sequences (ex. fatty acid synthase and cgd5_2180), and telomeric sequences of *Cryptosporidium*. Sequences of 18 contigs (#303, 357, 392, 415, 416, 436, 492, 503, 521, 524, 529, 537, 542, 543, 551, 562, 563, and 564) had no similarity to any published *Cryptosporidium* sequences, and one contig (#392) had 98-100% sequence similarity to *Bacteroides fragillis* plasmids from humans (AB646744 and U25716). Similar observations were made for TU502. In addition, the 547 bp at the 5′ end of contig AAEL01000108 (19,113 bp) had 98% sequence similarity to cgd3_530 on chromosome 3, while the remaining part of the sequence mapped to chromosome 8. Similarly, the 5′ (15,709-bp) region of contig AAEL01000024 (36,266 bp in length) mapped to chromosome 7, the 3′ region (nucleotides 25,790-36,266) mapped to chromosome 2, while the middle region containing the rRNA unit mapped to chromosomes 1, 2, 7, and 8.

In contrast, most of the unmapped contigs from IaA28R4 specimens 30976 and 33537 had non-*Cryptosporidium* sequences. For example, the largest 100 unmapped contigs (16,411-138,945 bp) from specimen 33537 were 99-100% similar to the genome (CP006252) of the enterobacteria *Serratia liquefaciens*, with the exception of contig 0018 (94,132 bp), which was from its plasmid. As the genome of *S. liquefaciens* is about 5.2 Mb, the 1,464 contigs of 14,065,231 bp from specimen 33537 were from the combined *C. hominis* and *S. liquefaciens* genomes, with all *S. liquefaciens* contigs positioned behind the mapped *Cryptosporidium* sequences (Additional file 1: Figure S1). Evidence of contamination from several bacterial species was present in data from specimen 30976, as the 6,140 contigs totaled 22.13 Mb, which is larger than the combined genomes of *C. hominis* and one bacterial species. BLAST analysis of contigs indicated that ~28% of the total nucleotides were from members of Enterobacteriaceae and 8% from Bacteroidaceae. The 20 largest unmapped contigs (88,676-515,888 bp) had 75-85% sequence similarities to genomes of members (*Serratia, Yersinia, Klebsiella, E. coli, Salmonella*, etc.) of Enterobacteriaceae, except for one (contig #51), which had a 98% sequence similarity to a 21,307 bp region of an uncultured organism from the human gut (GQ873945).

### Sequence similarity to published *C. parvum* genome and physical characteristic of *C. hominis* genomes

**Figure 2:**
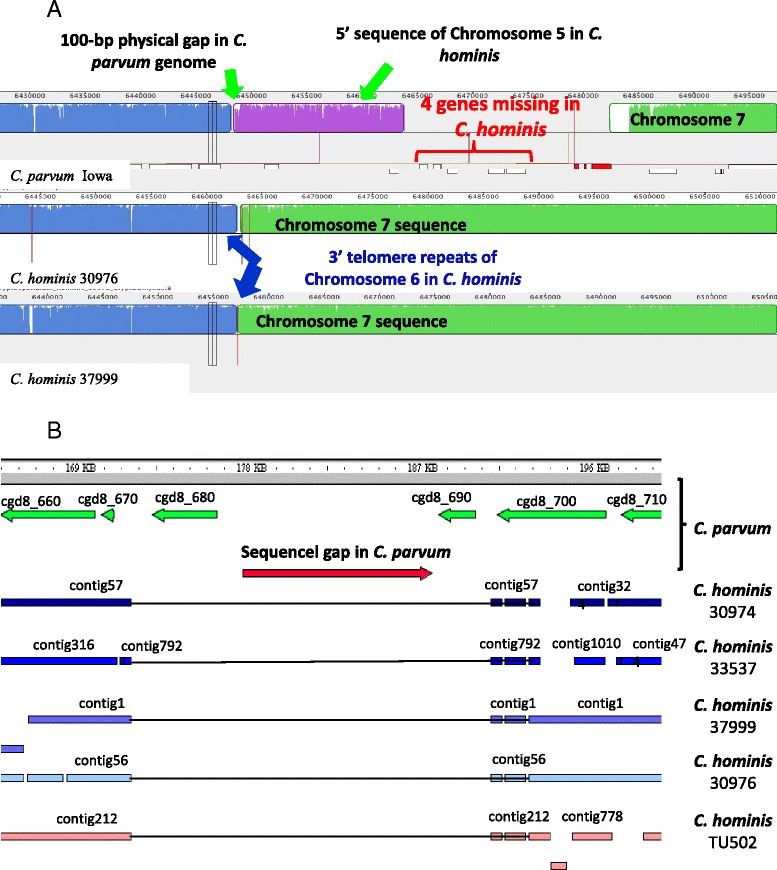
Deletion of genes in *Cryptosporidium hominis* genomes in comparison with *Cryptosporidium parvum*. **A**. Deletion of four genes (cgd6_5480, cgd6_5490, cgd6_5510, and cgd6_5520) at the 3′ end of chromosome 6 (probably should be the 5′ end of chromosome 5) in *C. hominis*. **B**. A major 19,048-bp deletion in *C. hominis* genome in chromosome 8, including the cgd8_680 and cgd8_690 genes. Note the ~10 kb sequence gap in *C. parvum*.

The genomes of specimens 30974, 30976, 33537, 37999 and TU502 had 96.90%, 96.87%, 97.0%, 96.86%, and 96.84% sequence similarities to the *C. parvum* IOWA genome in the mapped regions, respectively (Table 2). The alignment of whole genome sequences generated by Mauve showed near complete sequence synteny of the four *C. hominis* genomes to the published *C. parvum* reference genome, which is the only complete *Cryptosporidium* genome available for comparison. Some possible inversions and translocations of sequence fragments were seen in chromosomes 2, 4, 5 and 6 in the two almost fully sequenced *C. hominis* genomes from specimens 30976 and 37999 (Figure 1A). However, these inversions and translocations all occurred in sequence gap regions of the reference *C. parvum* genome. For example, specimens 30976 and 37999 generated sequences that cover the sequence gap in chromosome 2 of the *C. parvum* genome and have ~4,600-bp extra sequences at the 5′ end of the fragment, with telomeric repeats (TTTAGG) (Figure 1B). The inversions of sequences in chromosomes 4 and 5 also happened around sequence gaps in the *C. parvum* IOWA genome (Figure 1C). Near the 3′ end of chromosome 5, the large *C. hominis* contigs in 30976 and 37999 that are upstream of the two small scaffolds (coding for cgd5_4510 to cgd5_4610) in the *C. parvum* IOWA genome both end with telomeric repeats (data not shown). Similarly, the large *C. hominis* contigs in 30976 and 37999 that are upstream of the small scaffold (coding for cgd6_5460 to cgd6_5520) at the 3′ end of chromosome 6 in the *C. parvum* IOWA genome both end with telomeric repeats. In addition, one or two of the genes coded by the *C. parvum* scaffold (cgd6_5460 for specimen 37999 and cgd6_5460 and cgd6_5470 for specimen 30976) are located at the 5′ end of chromosome 5 in *C. hominis*, which has telomeric repeats at the 5′ end (Figure 2A). Most of the remaining genes are missing in *C. hominis*, except for cgd6_5500, whose ortholog is present in *C. hominis* in an unknown chromosome together with the ortholog of cgd5_4600. Although the genome sequences of specimens 30974 and 33537 were more fragmented, the same inversion of scaffolds was seen in chromosome 5 (Additional file 1: Figure S1).

Most of the missing sequences in the sequenced genomes were in the telomeric regions or low sequence complexity areas of the eight chromosomes of *C. parvum*, and generally occurred in the two 454-sequenced and the published *C. hominis* genomes. Half of the ten sequence gaps (unsequenced regions present in clones) in the *C. parvum* genome were missing in the 454-sequenced and the published *C. hominis* genomes (data not shown). The two Illumina-sequenced genomes, however, fully covered most of the ten sequence gaps in the *C. parvum* IOWA genome (Table 3). Most of the sequences generated from *C. hominis* specimens 30976 and 37999 were longer than the estimated length of sequence gaps in the *C. parvum* IOWA genome, with the noticeable exception of the ~10,000-bp sequence gap in *C. parvum*, which is not present in all five *C. hominis* genomes (Table 3). This region was fully covered by contig 11 of 30976, contig 2 of 37999, contig 0057 of 30974, contig 0792 of 33537, and contig 212 (AAEL01000212) of TU502 (Figure 2B). In *C. hominis*, the ~100 bp sequence downstream of the deletion is almost identical to the beginning sequence of the insert and the immediate sequence downstream of the insert in *C. parvum*. This might have contributed to the large deletion in the *C. hominis* genome. The size of the deletion was 19,048 bp if the sequence gap in *C. parvum* was indeed 10,000 bp.

**Table 3:** **Coverage of two Illumina-sequenced** ***Cryptosporidium hominis*** **genomes in sequence gaps of the published** ***C. parvum*** **IOWA genome**

**Chromosome**	**Gap in** ***C. parvum*** **IOWA (bp)**	**Sequence length in** ***C. hominis*** **specimen (bp)**	
		**37999**	**30976**
2	100*	1481	1481
3	500	5582	5576
4	100 (1^st^)*	>458	1450
4	100 (2^nd^)*	2937	2714
5	100 (1^st^)*	14,926	14,929
5	100 (2^nd^)*	1788	1311
5	1,000 (3^rd^)	Not covered	467
6	2,500	245	245
6	100*	>538 (ending with telomeric repeats)	>857 (ending with telomeric repeats)
8	10,000	19,048 bp deletion spanning entire gap	19,048 bp deletion spanning entire gap

*Regions where inversions and translocations of sequences occurred in sequenced *C. hominis* genomes.

### *C. parvum*- and *C. hominis*- specific sequences

In addition to the large 19,048-bp deletion, which contained the *C. parvum*-specific cgd8_680 and cgd8-690 genes and other potential genes in the ~10,000-bp sequence gap, the comparative genomic analysis identified several other deletions in the *C. hominis* genome (Table 4), including those mentioned above at the 3′ end of chromosomes 5 (containing orthologs of cgd5_4580, cgd5_4590, and cgd5_4610) and 6 (containing orthologs of cgd6_5480, cgd6_5490, cgd6_5510, and cgd6_5520). Most of the *C. parvum*-specific genes were members of multicopy gene families. Thus, cgd5_4580, cgd5_4590, cgd5_4600, cgd5_4610, cgd6_5480, and cgd6_ 5490 in *C. parvum* are genes of the *Cryptosporidium* telomeric MEDLE family of secreted proteins, all with signal peptides and similar sequences. As mentioned above, these genes are located in tandem in telomeric regions of these two chromosomes, but *C. hominis* has only one such gene, Chro.50507 or the ortholog of cgd5_4600. Likewise, cgd6_5510 and cgd6_5520 both code for telomeric insulinase-like proteases with signal peptides. Members of the insulinase-like proteases all have very different nucleotide sequences, and both *C. parvum* and *C. hominis* have 11 such genes in tandem near the 3′ end of chromosome 3. Thus, *C. hominis* lacks two of the subtelomeric genes at the 5′ end of chromosome 5 as well as five copies of the *Cryptosporidium* telomeric MEDLE family of secreted proteins in chromosomes 5 and 6. The *C. parvum-*specific nature of cgd6_5510 (also known as ZPT) was previously known [28].

**Table 4:** **Species-specific genes in genomes of** ***Cryptosporidium parvum*** **and** ***C. hominis***

**Chromosome**	**Length (bp)**	**Genes**	**Specificity**
8	19,048	cgd8_680, cgd8_690 and other potential genes in 10,000 bp sequence gap	*C. parvum*
6	15,314	cgd6_5480, cgd6_5490, cgd6_5510, cgd6_5520	*C. parvum*
5	5,620	cgd5_4580, cgd5_4590, cgd5_4610	*C. parvum*
3	~4800	Chro.50011	*C. hominis*

Notes:

1. cgd5_4580, cgd5_4590, cgd5_4600, and cgd5_4610: four genes with similar sequences at the 3′ end of chromosome 5 in *C. parvum*, all called telomeric MEDLE family of secreted proteins. *C. hominis* has only one such gene here (Chro.50507, the ortholog of cgd5_4600).

2. cgd6_5480 and cgd6_ 5490: two genes of the telomeric MEDLE family of secreted proteins with similar sequences at 3′ end of chromosome 6 in *C. parvum. C. hominis* has no such gene here. The two genes have sequences similar to the four genes above. This fragment and cgd6_5510 (ZPT) and cgd6_5520 below are located at the 5′ end of chromosome 5 in the *C. hominis* genomes sequenced. *C. hominis* specimen 37999 does not appear to have the ortholog for cgd6_5470, although 30976 clearly has it. Ortholog of cgd6_5500 is apparently translocated to an unknown chromosome in *C. hominis*, downstream of the ortholog of cgd5_4580.

3. cgd6_5510 (ZPT) and cgd6_5520: telomeric insulinase-like protease with a signal peptide (the two genes have very different sequences). *C. parvum* has 11 such genes near 3′end of chromosome 3.

4. cgd8_680: a large low complexity protein with repeats. cgd8_690: a signal peptide containing protein with 2 *Cryptosporidium*-specific paralogs (cgd8_660 and its ortholog chro.80081).

**Figure 3:**
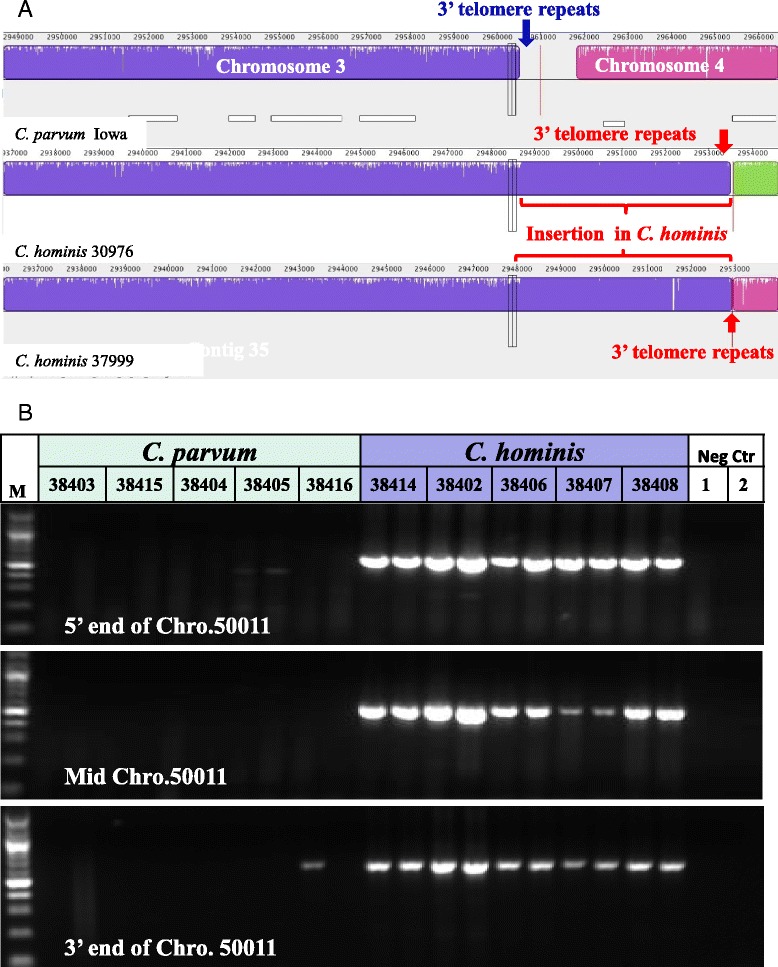
*Cryptosporidium hominis*-specific nature of Chro.50011. **A**. Insertion of ~4,860 bp containing the Chro.50011 gene at the 3′ end of chromosome 3 in *C. hominis*. **B**. Confirmation of the absence of the ortholog of Chro.50011 in four specimens of *C. parvum* by PCR analysis of three regions of the Chro.50011 gene. The faint band in PCR analysis of the 3′ end of the gene in *C. parvum* specimen 38416 produced a nucleotide sequence identical to Chro.50011 in *C. hominis*.

The comparative genomic analysis further identified several large contigs not found in the published *C. parvum* IOWA genome, including contigs AAEL01000413, AAEL01000717, and AAEL01000728 in the published TU502 genome. Contig AAEL01000413 is 6,056 bp in length (29% GC) and has 260 copies of telomeric sequence TTTAGG at its 3′ end. It mapped to contigs 442 and 290 of specimen 30974 and contigs 1743, 1586, and 768 of specimen 33537, and was fully covered by contig 82 of specimen 30976 and contig 35 of specimen 37999. It codes at nucleotides 1,798-3,267 for the hypothetical protein Chro. 50011 with RS/HS repeats at the carboxyl end. The coding region was fully covered in all *C. hominis* genomes sequenced in this project. Sequence alignment indicates that the gene is located at the 3′ end of chromosome 3, with the entire insertion (4,795 bp in 30976 and 4,830 bp in 37999, excluding telomere repeats) all ending with copies of the telomere repeat sequence TTTAGG (Figure 3A). As the 3′ end of chromosome 3 of *C. parvum* also had the telomeric repeats, this insertion appears valid. The coding region is not present in *C. parvum* EST library data and five MS/MS peptide libraries deposited in CryptoDB (http://cryptodb.org/cryptodb/). PCR amplification of DNA from five *C. parvum* and *C. hominis* specimens each confirmed its presence in only *C. hominis* (Figure 3B).

In contrast, contig AAEL01000728 is 2,277 bp in length (23% GC) and mapped to contig 257 of specimen 30974 and contigs 1238, 1367, and 1487 of specimen 33537. It is located in chromosome 5 of specimens 30976 (contig_6) and 37999 (contig_1), and within a sequence gap area in the *C. parvum* IOWA genome. PCR analysis using primers based on the AAEL01000728 sequence amplified DNA of *C. hominis, C. parvum*, and *C. andersoni*, with the sequences from *C. parvum* and *C. hominis* differing from each other by two nucleotides in the 413-bp region, and from *C. andersoni* having 97% sequence similarity to nucleotide 19,798-20,202 of XM_002142452 (coding for a large hypothetical protein CMU_010870) from *C. muris* (data not shown). Contig AAEL01000717, which contains the sensor histidine kinase gene (Chro.00003, nucleotide 673–2,319), was probably not of *Cryptosporidium* origin. It is 2,333 bp in length had a 66% GC content. It has no equivalents in the published *C. parvum* and *C. muris* genomes and *C. hominis* genomes sequenced in the present study, but has a 77% sequence similarity to the sensor histidine kinase gene of *Rhizobium etli* (nucleotides 1,334,700-1,334,429 of CP001074). PCR primers based on this sequence did not amplify DNA of *C. parvum* or *C. hominis* (data not shown).

### Sequence similarity to published *C. hominis* genomic data

**Figure 4:**
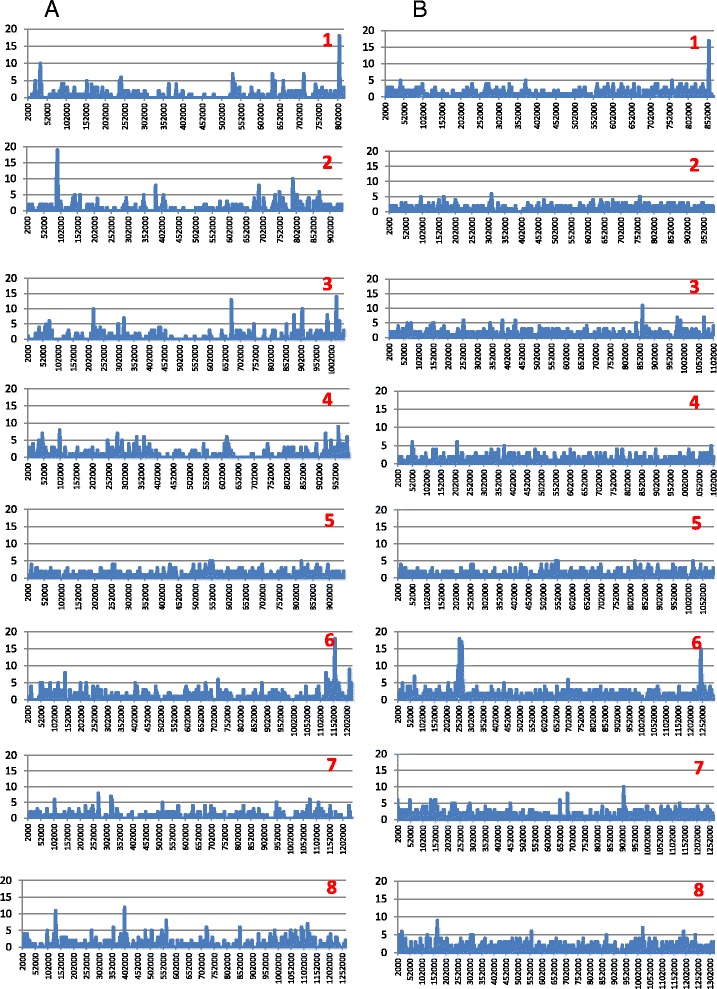
Distribution of SNPs in *Cryptosporidium hominis* genome by chromosome. The number of SNPs in a sliding window of 2,000 bp with 200 bp steps across each of the eight chromosomes is shown. **A**. Sequence divergence between specimen 30976 of the IaA28R4 subtype and the published isolate TU502 of the IaA25R3 subtype. **B**. Sequence divergence between specimen 37999 of the IbA10G2 subtype and specimen 30976 of the IaA10G2 subtype.

The genomes of specimens 30974 (IbA10G2), 30976 (IaA28R4), 33537 (IaA28R4), and 37999 (IbA10G2) had 99.78%, 99.83%, 99.83%, and 99.72% sequence similarities to the published *C. hominis* genome of TU502 (of the IaA25R3 subtype), respectively (Table 2). Mapping of Illumina reads from specimens 30976 and 37999 to the contigs of the published *C. hominis* TU502 genome indicated that single nucleotide polymorphisms (SNPs) were distributed across all eight chromosomes of the genome. A few loci on several chromosomes, however, had higher sequence polymorphisms (Figure 4A, Additional file 2: Figure S2). Most of the highly polymorphic loci occurred in genes coding for mucins (orthologs of cgd2_430, cgd2_440, cgd2_450, cgd3_720, and cgd6_1080 or gp60), other secreted proteins with signal peptides (orthologs of cgd1_150, cgd1_3810, cgd3_3430, cgd6_1030, and cgd6_5270), and proteases (orthologs of cgd3_4260 and cgd6_60) (Table 5). A CryptoDB search of transcriptome data generated from an *in vitro* culture of *C. parvum* previously by real-time PCR [29] indicated that most of the genes are differentially expressed at various developmental stages.

**Table 5:** **Highly polymorphic loci in** ***Cryptosporidium hominis*** **genomes**

**Locus**	**Contig in 30976**	**SNP/kb (30976 vs Tu502)**	**Gene in** C. hominis*	**Ortholog in** C. parvum*	**Annotation**
chr1_var1	contig_5	5.0	Chro.10024	cgd1_150	Hypothetical protein with a signal peptide
chr1_var2	contig_5	9.0	Chro.10427	cgd1_3810	Conserved hypothetical protein with a signal peptide
chr2_var1	contig_32	9.5	Chro.20050-52	cgd2_430-450	Mucin glycoprotein with a signal peptide
chr2_var2	contig_20	5.0	Intergenic downstream of Chro.20394	Intergenic downstream of cgd2_3690	WD repeat protein (cgd2_3690)
chr3_var1	contig_59	5.0	Intergenic downstream of Chro.30096	Within cgd3_720	Very large mucin with a signal peptide
chr3_var2	contig_31	6.5	Chro.30315	cgd3_2770	Hypothetical conserved protein
chr3_var3	contig_293 + contig_255	7.0	Chro.30479	cgd3_4260	Insulinase-like protease
chr6_var1	contig_3	9.0	Chro.60606	cgd6_5270	Hypothetical protein with a signal peptide
chr8_var1	contig_11	5.5	Chro.80070	cgd8_550	Large uncharacterized protein
chr8_var2	contig_2	6.0	Chro.80189	cgd8_1610	Sacsin-like HSP90 chaperone domain

*Additional polymorphic genes identified by comparative analysis of other isolates with *C. hominis* TU502: Chro.60016 (ortholog of cgd6_60), and Chro.60138 (ortholog of cgd6_1080).

### Sequence similarity among sequenced *C. hominis* genomes and occurrence of genetic recombination

The genomes of the four *C. hominis* specimens sequenced in this study were similar to each other, except for a subtelomeric region at the 3′ end of chromosome 1 and three regions on chromosome 6. This was supported by SNP analyses through both mapping of Illumina reads to assembled contigs (Figure 4B) and direct comparison of sequence alignments of assembled contigs. At the 3′ end of chromosome 1 (within Chro.10427 or the ortholog of cgd1_3810), two types of sequences were seen among the specimens sequenced in this study: one from the two IbA10G2 specimens (30974 and 37999) and another from the two IaA28R4 specimens (30976 and 33537). Both were very divergent from the sequence in TU502 (Figure 4, Additional file 2: Figure S2). In contrast, at the three loci in chromosome 6, the sequence polymorphism was biallelic, with each genome showing one of the two types of nucleotide sequences, including the reference *C. hominis* genome (Table 6). Thus at the 5′ end of the chromosome (containing Chro.60016, the ortholog of cgd6_60, coding for a protease), all specimens except for specimen 30974 had sequence identical to the published sequence from TU502 (Additional file 3: Figure S3). At the gp60 locus (Chro.60138, the ortholog of cgd6_1080: a well-known subtyping locus for *Cryptosporidium*), specimens 30974 and 37999 had the Ib type sequence whereas others, including TU502 had the Ia type sequence. Similarly at the 3′ end of the chromosome downstream of Chro.60606 (the ortholog of cgd6_5270), specimens 30974 and 37999 had sequences similar to the published sequence from TU502, whereas specimens 30976 and 33537 had a different type of sequence (Figure 4, Additional file 2: Figure S2, Table 6). The breakpoints for the three genetic recombination areas occurred at intergenic regions upstream and downstream of cgd6_60 (ortholog: Chro.60016), upstream of cgd6_1000 (ortholog: Chro.60130) and downstream of cgd6_1100 (ortholog: Chro.60142), and upstream of cgd6_5240 (ortholog: Chro.60603) and downstream of cgd6_5320 (its ortholog in *C. hominis* is unnamed) for the three regions, respectively. Because of the occurrence of genetic recombination, the two IbA10G2 specimens (30974 and 37999) sequenced in the study had different types of sequence at the 5′ end of chromosome 6 (Table 6).

**Table 6:** **Genetic recombination in chromosome 6 of two virulent** ***Cryptosporidium hominis*** **subtypes**

**Specimen (subtype)**	**Sequence characteristics**		
	**5′ end (cgd6_60)**	**gp60 area (cgd6_1000-cgd6_1100)**	**3′ end (cgd6_5240-cgd6_5320)**
30974 (IbA10G2)	IbA10G2	IbA10G2	IaA25R3
37999 (IbA10G2)	IaA25R3*	IbA10G2	IaA25R3
30976 (IaA28R4)	IaA25R3	IaA28R4	IaA28R4
33537 (IaA28R4)	IaA25R3	IaA28R4	IaA28R4
TU502 (IaA25R3)	IaA25R3	IaA25R3	IaA25R3

*15/16 SNPs at the 3′ end of cgd6_60 are unique in 37999.

### Intra-specimen sequence diversity at the trinucleotide repeat region of gp60

Because of a recent report on intra-specimen genetic heterogeneity seen in Illumina sequencing of a PCR-WGA product from a *C. parvum* specimen [30], we examined intra-specimen sequence diversity at the trinucleotide repeat region of the gp60 gene in the four *C. hominis* specimens sequenced in this study. In the specimens sequenced by using 454 technology, 205 and 310 sequence reads mapped to the gp60 gene for specimens 30974 and 33537, respectively. Among them, 78 and 59 reads had sequences fully covering the entire trinucleotide repeats for the IbA10G2 and IaA28R4 subtypes, respectively. No intra-specimen sequence diversity was seen (Additional file 4: Figure S4, Additional file 5: Figure S5). Similarly, 2,781 and 5,576 sequence reads mapped to the gp60 gene in specimens 37999 and 30976 sequenced by using Illumina, respectively. Among them, 73 and 30 reads had sequences fully covering the entire trinucleotide repeats for the IbA10G2 and IaA28R4 subtypes, respectively. No intra-specimen diversity was seen in specimen 37999, whereas in 30976 (of the IaA28R4 subtype), 28 reads had 28 copies of the TCA repeat, one had 27 copies of the TCA repeat, and one had 29 copies of the TCA repeat (data not shown).

## Discussion

### Genome similarity between *C. hominis* and *C. parvum*, gene deletions, and species-specific genes

Results of this study have confirmed the genetic similarity between the almost fully sequenced *C. parvum* and *C. hominis* genomes. The genomes of the two species are nearly 97% similar in nucleotide sequences, with complete synteny in gene organization. This is similar to the previous conclusion based on comparison of the fully assembled genome of the *C. parvum* IOWA isolate and the more fragmented genome from the *C. hominis* TU502 isolate [24,25]. Some potential genetic rearrangements in several chromosomes were observed in the current study, but they all occurred in the ten sequence gaps and several sequence ambiguity areas in the reference *C. parvum* genome. As there are no HAPPY maps and genomic libraries with large inserts for *C. hominis*, the observations on genome organization of *C. hominis* need to be supported by PacBio sequencing. Nevertheless, comparative genomic analysis in this study has identified several major deletions and one insertion in *C. hominis*, which were overlooked in previous studies probably because of the fragmented nature of the published *C. hominis* genome. The significance of these gene insertions and deletions (indels) is not clear. Because of the high sequence similarity in most genes between *C. parvum* and *C. hominis*, these major indels could potentially be responsible for some biological differences between *C. parvum* and *C. hominis*.

Gene duplication and interallelic recombination could contribute to the gene expansion and losses seen between *C. parvum* and *C. hominis* genomes. Most of the genes deleted in the *C. hominis* genome are members of multigene families and have paralogs nearby. Thus, of the six MEDLE family of secreted protein genes possibly present in tandem in *C. parvum* (cgd5_4580, cgd5_4590, cgd5_4600, cgd5_4610, cgd6_5480, and cgd6_ 5490), only one, the ortholog of cgd5_4600, is present in *C. hominis*. Similarly, two genes (cgd6_5510 and cgd6_5520) that code for insulinase-like are absent in *C. hominis*. The subtelomeric locations of these genes facilitate the expansion and deletions of multicopy genes by interallelic recombination. Sequence homology is probably also involved in the loss of cgd8_680 and cgd8_690 orthologs in chromosome 8 of *C. hominis*, as the ~100 bp region upstream of the fragment containing the two genes and the ~100 bp region downstream of the fragment have almost identical sequences. The sequence homology in two nearby regions could have resulted in the deletion of the two genes in *C. hominis* during species evolution. As cgd8_690 is a paralog of cgd8_660 and has some sequence similarity to the 5′ end of cgd8_670, this gene loss in chromosome 8 of *C. hominis* also involves a multigene family. In compact apicomplexan genomes with mostly single copy genes, members of multigene families usually play very important biological functions [31,32]. The function of the MEDLE family of secreted proteins in apicomplexan parasites has not been examined. However, insulinase-like proteases have been shown recently to be rhoptry or microneme-associated in *Toxoplasma gondii* and are probably involved in cell invasion [33,34]. Indeed, both cgd6_5510 and cgd6_5520 have peak expression during the invasion process. The expression of cgd6_5480 and cgd6_ 5490 in *C. parvum* may also be developmentally regulated, as they showed identical expression patterns in *in vitro* culture [29]. As sequence differences in non-coding regulatory elements can also affect the timing or expression levels of invasion-associated proteins, more studies are needed to determine whether the duplications of MEDLE and insulinase genes are indeed the cause of the host expansion of in *C. parvum*.

Compared to the deletion of at least nine genes, *C. hominis* appears to have only one unique gene that is absent in *C. parvum*. This gene, Chro.50011, is located at the 3′ end of chromosome 3 instead of the original annotation at the 5′ end of chromosome 5 (Figure 2A). It codes for a 489 aa hypothetical protein that contains RS and HS repeats at the carboxyl end, and has recently been identified as a *C. hominis*–specific gene, Chos-1, by Bouzid and colleagues [35]. Although the function of the protein is not clear, it has been suggested that this protein is a member of a new *Cryptosporidium*-specific protein family that are candidate mediators of host specificity and virulence [35]. It remains to be determined whether the *C. hominis* genome codes for additional species-specific genes in areas of the ten sequence gaps in the *C. parvum* IOWA genome.

### Sequence similarity among *C. hominis* genomes and genetic recombination in virulent *C. hominis* subtypes

As expected, much higher genetic similarity is present among *C. hominis* genomes. The four *C. hominis* specimens sequenced in this study had whole genome sequences that are 99.72-99.83% similar to the published *C. hominis* genome from TU502. Genes coding for some secreted proteins (especially mucins) and proteases contribute more to the sequence differences than others, suggesting they are under selection and therefore may serve as good targets for the development of diagnostic tools and intervention measures. For example, some of the polymorphic mucin genes such as cgd2_430 (Mucin5) and cgd6_1080 (gp60) are well known targets of host immune responses [36,37] and have been used widely in subtyping *C. parvum* and *C. hominis* [10]. Proteases (especially cysteine proteases) and protein kinases have been recently shown to play important roles in cell invasion of *Cryptosporidium* and thus have been used as common targets in the development of therapeutic treatments [38-41].

In contrast to the relatively high nucleotide sequence differences between the genomes sequenced in this study and the published *C. hominis* TU502 genome, the genomes of four specimens from two virulent *C. hominis* subtypes (IbA10G2 and IaA28R4) in the United States are very similar to each other except for the 3′ end of chromosome 1 and three areas in chromosome 6. In particular, chimeric sequences were seen in chromosome 6 (Table 6), indicating the occurrence of genetic recombination in the two subtypes. One of the three areas with genetic recombination is where gp60 (cgd6_1080) is located, a locus widely known for its extremely high sequence diversity and occurrence of genetic recombination [42]. Recently, population genetic analyses of chromosome 6 sequences have shown the exclusive occurrence of genetic recombination in the virulent *C. hominis* subtypes IbA10G2 and IaA28R4, especially around gp60 [43,44]. It was postulated that the fitness of the two subtypes as a result of genetic recombination was likely responsible for the wide dissemination of IbA10G2 around the world and the emergence of IaA28R4 in the United States. The two IbA10G2 specimens sequenced in this study also differ from each other at the 5′ end of chromosome 6, especially in the ortholog of cgd6_60 (coding for a protease) as a result of genetic recombination. It was previously shown by MLST analysis of chromosome 6 that IbA10G2 specimens from different areas are genetically different [45]. Although the two IaA28R4 specimens sequenced in this study are mostly identical, data from a recent population genetic study of IaA28R4 specimend in the United States suggest that there are at least two origins of the subtype [44]. Therefore, multiple genetic recombination events are probably involved in the evolution of both IbA10G2 and IaA28R4 and are likely responsible for the observed emergence of the same virulent gp60 subtypes in different geographical locations in response to selection pressure [46]. The occurrence of genetic recombination in virulent *C. hominis* subtypes also suggests that the widely used gp60-based typing alone is insufficient in molecular epidemiologic characterizations of field specimens, as pointed out previously [46]. Therefore, the use of MLST and other multilocus subtyping tools can provide new insights into the transmission of *Cryptosporidium* spp. [44,47-49]. As expected, the three loci in chromosome 6 where genetic recombination occurs, cgd6_60, cgd6_1080, and cgd6_5270 (coding for a hypothetical protein with a signal peptide and paralogs) are all highly polymorphic in the present study. The biological functions of proteins coded by cgd6_60 and cgd6_5270 thus should be studied.

## Conclusion

In conclusion, this comparative genomic analysis has revealed some unique genetic differences between *C. parvum* and *C. hominis* and identified some multigene families that can potentially contribute to differences in host specificity of the two closely related species. It has further supported the potential role of genetic recombination in the emergence and evolution of virulent *C. hominis* subtypes. Improvements in knowledge in these two areas are still hampered by the lack of genomic studies of other *Cryptosporidium* species of significant public health and economic importance, the incompleteness of the reference *C. parvum* and *C. hominis* genomes, and poor understanding of the functions of the thousands of hypothetical proteins in *Cryptosporidium* genomes and regulatory elements in non-coding areas. With the increased recognition of the importance of cryptosporidiosis in pediatric health in developing countries [3], common occurrence of large waterborne outbreaks in industrialized nations [15,16,50], and a major increase in cryptosporidiosis incidence in the United States in recent years [6,8,9], more effort should be directed toward studies on functional genomics and the basic biology of *Cryptosporidium* spp. [51].

## Methods

### *Cryptosporidium* specimens

Four *C. hominis* specimens were used in whole genome sequencing in the study: specimens 30974 and 37999 of the IbA10G2 subtype and 30976 and 33537 of the IaA28R4 subtype. Specimen 30974 was collected from a patient from a cryptosporidiosis outbreak in July 2010 in Columbia, South Carolina associated with a splash pad that had problems with filtration and chlorination. Testing of filter backflush and stools from six patients all identified the presence of the *C. hominis* IbA10G2 subtype. Specimen 30976 was collected from a patient in a cryptosporidiosis outbreak in July 2010 in the St. Louis area in Illinois and Missouri associated with swimming pools and a water park. Testing of nine patient specimens identified the occurrence of *C. hominis* IaA28R4 in seven patients, IaA24R4 in one patient, and IdA15G1 in another patient. Specimen 33537 was collected from a patient from a cryptosporidiosis outbreak in July 2011 in Walsenburg, Colorado associated with a waterpark that had problems with the chlorinator. Testing of filter backflush and stools from five patients identified IaA28R4 in all. Specimen 37999 was collected from a sporadic cryptosporidiosis patient in Twin Falls, Idaho in September 2012. All stool specimens were collected fresh from symptomatic patients and stored in 2.5% potassium dichromate at 4°C prior to being used in *Cryptosporidium* oocyst isolation for whole genome sequencing within 6 months. *Cryptosporidium* species and subtypes were determined by PCR-RFLP analysis of the small subunit rRNA and sequence analysis of the 60 kDa glycoprotein (gp60) genes, respectively [17].

### Oocyst isolation and whole genome amplification

*Cryptosporidium* oocysts were isolated from stool specimens by discontinuous sucrose and cesium chloride gradients as previously described [52]. They were further purified by immunomagnetic separation using the Dynabeads Anti-*Cryptosporidium* kit (Invitrogen, Carlsbad, CA). After treating the purified oocysts with 10% commercial bleach on ice for 10 min and five cycles of freezing and thawing, DNA was extracted from them by using the Qiagen DNeasy Blood & Tissue Kit (Qiagen, Valencia, CA). Whole genome amplification (WGA) of the 25–100 ng of extracted DNA was conducted by using the REPLI-g Midi Kit (Qiagen). The quality of the WGA products was verified by sequencing BamHI-digested WGA products cloned into a pUC19 vector (Fermantas, Pittsburgh, PA). The sequencing was done by using the ABI BigDye Terminator v3.1 Cycle Sequencing Kit on an ABI3130 Genetic Analyzer (Applied Biosystems, Foster City, CA).

### 454 and Illumina sequencing and *de novo* contig assembly

The WGA products from specimens 30974 and 33537 were sequenced with 454 technology on a GS-FLX Titanium System (Roche, Branford, CT) by using approximately 1 μg of DNA for library construction and following standard Roche library protocols, with an average insert size of 600 bp. One full PTP plate was used in the analysis of each specimen. The sequence reads from each run were assembled using Newbler in the GS De Novo Assembler (http://www.454.com/products/analysis-software/) with the default settings.

The WGA products from specimens 30976 and 37999 were used to generate Illumina TruSeq (v3) libraries (average insert size: 350 bp) and sequenced 100×100 bp paired-end on an Illumina Genome Analyzer IIx (Illumina, San Diego, CA). The sequence reads with a minimum quality of 20 were trimmed by using CLC Assembly Cell 4.1.0 (http://www.clcbio.com/products/clc-assembly-cell/). The data were then assembled with default parameters and a minimum contig length of 500 bp, with scaffolding using paired-end data.

### Comparative genomic analyses

For comparisons of sequences at the genome level, contigs of each specimen were aligned with reference sequences of the near complete genome of the *C. parvum* IOWA isolate (version AAEE00000000.1) and the 1,422 contigs of the *C. hominis* TU5205 isolate (version NZ_AAEL00000000.1) using Nucmer, a tool in MUMmer 3.23 (http://mummer.sourceforge.net/) [53]. Multiple genome alignments were also constructed by using the progressive alginment algorithm of the Mauve 2.3.1 (http://asap.genetics.wisc.edu/software/mauve/) with default options [54]. In-house perl scripts were developed to calculate the average nucleotide identities. For the detection of SNPs, Fastqc 0.10.0 (http://www.bioinformatics.babraham.ac.uk/projects/fastqc/) was used for the QC analysis of Illumina sequence reads, and PRINSEQ 0.20.3 (http://prinseq.sourceforge.net/) [55] was used to remove low quality reads, with a min_qual_mean setting of 20 and min_len of 65. Reads were then aligned to reference sequences by using Bowtie 0.12.7 (http://bowtie-bio.sourceforge.net/index.shtml) [56]. The resulting SAM files were processed, sorted and duplicates were removed by using Picard 1.126 (http://broadinstitute.github.io/picard/). The mpileup in SAMtools (http://samtools.sourceforge.net/) was finally used to create the pileup file for SNP variant calls using the mpileup2snp in VarScan 2.3.7 (http://varscan.sourceforge.net/) [57]. Default parameters for VarScan were used except that min-avg-qual was set to 30.

### PCR verification

As the comparative genomic analysis had identified some nucleotide sequences (AAEL01000413, AAEL01000728, and AAEL01000717) in the published *C. hominis* that had not been seen in the published *C. parvum* genome, primers were designed based on these sequences to verify the source of these sequences by PCR (Additional file 6: Table S1). Five specimens each of *C. parvum* and *C. hominis* were used in PCR analysis of each target. In addition, two *C. andersoni* specimens were used in confirmation of *Cryptosporidium*-origin of contig AAEL01000728. Each specimen was analyzed in duplicate nested PCR using 50 μl PCR mixture consisting of 1 μl (~100 ng) of extracted DNA or 2 μL of primary PCR products (in secondary PCR), 200 μM deoxynucleoside triphosphate, 1× PCR buffer (Applied Biosystems), 3.0 mM MgCl_2_, 5.0 U of Taq polymerase (Promega, Madison, WI), 100 nM primers, and 400 ng/μl of non-acetylated bovine serum albumin (Sigma-Adrich, St. Louis, MO). The primary and secondary PCR reactions were performed in a GeneAmp PCR 9700 thermocycler (Applied Biosystems) for 35 cycles of 94°C for 45 s, 55°C for 45 s, and 72°C for 60 s, with an initial denaturation (94°C for 5 min) and a final extension (72°C for 7 min). The secondary PCR products were sequenced in both directions using Sanger technology described above. Nucleotide sequences obtained were aligned with reference sequences downloaded from GenBank by using ClustalX (http://www.clustal.org/).

### NCBI BioProject No.

Nucleotide sequences generated from the project, including all SRA data and assembled contigs, were submitted to the NCBI BioProject under the accession number PRJNA252787.

### Ethics statement

The study was done on delinked residual diagnostic specimens. It was covered by Human Subjects Protocol No. 990115 “Use of residual human specimens for the determination of frequency of genotypes or sub-types of pathogenic parasites”, which was reviewed and approved by the Institutional Review Board of the Centers for Disease Control and Prevention (CDC). No personal identifiers were associated with the specimens at the time of submission for diagnostic service at CDC.

## Additional files

Additional file 1: Figure S1. Coverage of two Roche 454 sequenced genomes of *Cryptosporidium hominis* comparing to the published *C. parvum* (IOWA) and *C. hominis* (TU502) genomes. The eight chromosomes of *C. parvum* are numbered and assembled contigs are bordered by vertical red lines. The color blocks are conserved segments of sequences internally free from genome rearrangements, whereas the inverted white peaks within each block are sequence divergence between the reference *C. parvum* genome and *C. hominis* genome under analysis. Additional file 2: Figure S2. Sequence divergence between *Cryptosporidium hominis* IaA28R4 (specimen 30976) and IaA25R3 (isolate TU502) subtypes by chromosome. The number of the SNPs in a sliding window of 2,000 bp with 200 bp steps across each of the eight chromosomes is shown. Additional file 3: Figure S3. Polymorphic nucleotide sequences in mid part of the Chro.60016 (ortholog of cgd6_60, coding for a protease) gene in *Cryptosporidium hominis* specimens. Dots denote sequence identity to the reference TU502 sequence. Specimen 37999 also has different sequence at the 3′ end of the gene (not shown). Additional file 4: Figure S4. Lack of variation in sequence diversity in the trinucleotide repeat region in the gp60 gene of specimen 30974 of the *Cryptosporidium hominis* IbA10G2 subtype. Of 205 reads from 454 sequencing that mapped to gp60, 78 had complete sequence of the trinucleotide repeats, with no variation in repeat numbers. Dots denote sequence identity to the reference sequence, whereas dashes denote deletions of nucleotides. Additional file 5: Figure S5. Lack of variation in sequence diversity in the trinucleotide repeat region in the gp60 gene of specimen 33537 of the *Cryptosporidium hominis* IaA28R4 subtype. Of 310 reads from 454 sequencing that mapped to gp60, 59 had complete sequence of the trinucleotide repeats, with no variation in repeat numbers. Dots denote sequence identity to the reference sequence, whereas dashes denote deletions of nucleotides. Additional file 6: Table S1. Primers used in the PCR verification of possible *Cryptosporidium hominis*-unique nucleotide sequences.
