# Supplementary figures and images for: Comparative genomic analysis reveals occurrence of genetic recombination in virulent Cryptosporidium hominis subtypes and telomeric gene duplications in Cryptosporidium parvum
Source: BMC Genomics. 2015 Apr 18;16(1):320. doi: 10.1186/s12864-015-1517-1 (PMC4407392; doi:10.1186/s12864-015-1517-1)

## Slide 1
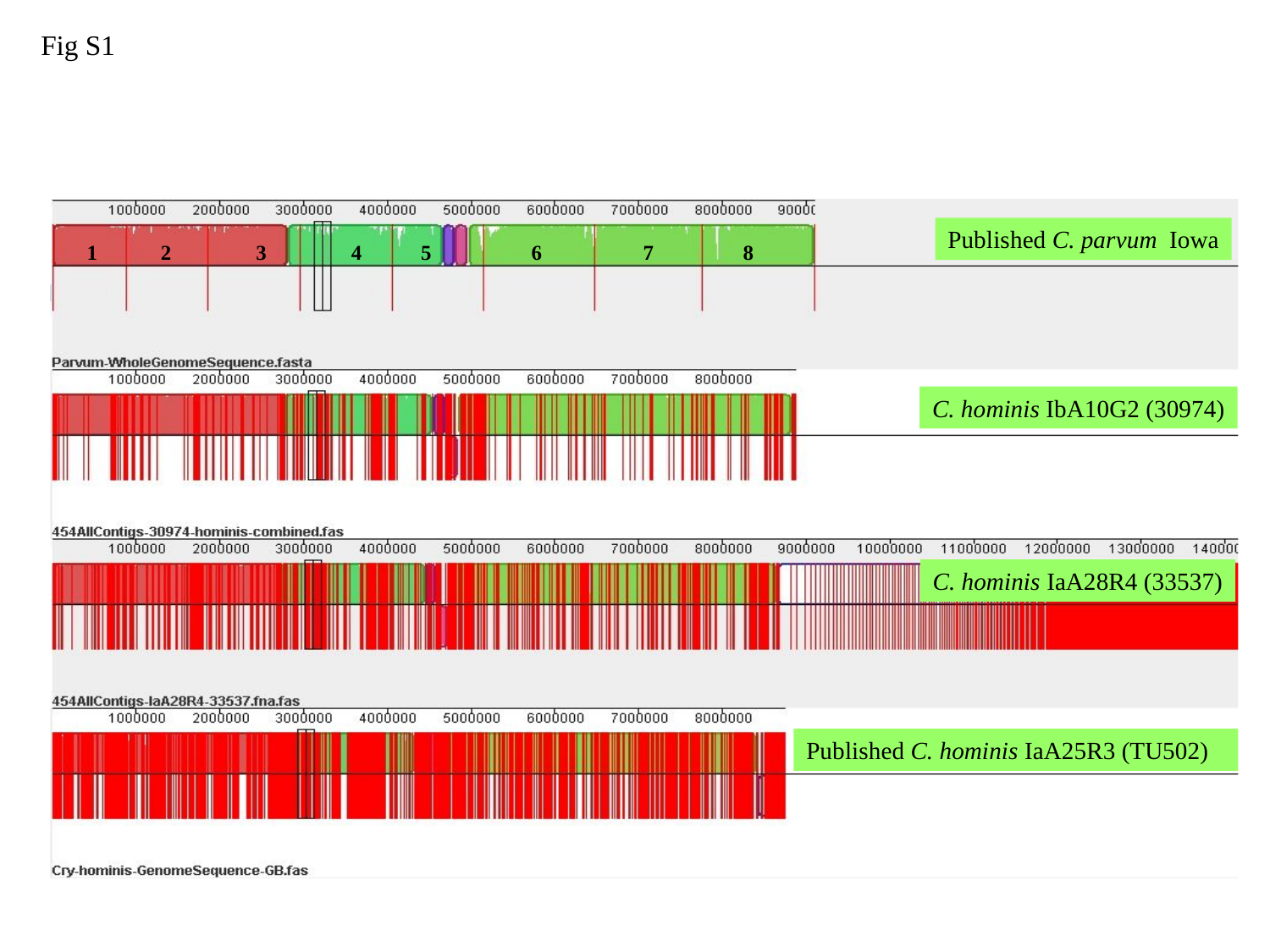

Fig S1
Published C. parvum Iowa
1
2
3
4
5
6
7
8
C. hominis IbA10G2 (30974)
C. hominis IaA28R4 (33537)
Published C. hominis IaA25R3 (TU502)

Supplement: Additional file 1: Figure S1. — Coverage of two Roche 454 sequenced genomes of Cryptosporidium hominis comparing to the published C. parvum (IOWA) and C. hominis (TU502) genomes. The eight chromosomes of C. parvum are numbered and assembled contigs are bordered by vertical red lines. The color blocks are conserved segments of sequences internally free from genome rearrangements, whereas the inverted white peaks within each block are sequence divergence between the reference C. parvum genome and C. hominis genome under analysis. [file 12864_2015_1517_MOESM1_ESM.pptx]
