# Supplementary material for: Comparative genomic analysis reveals occurrence of genetic recombination in virulent Cryptosporidium hominis subtypes and telomeric gene duplications in Cryptosporidium parvum
Source: BMC Genomics. 2015 Apr 18;16(1):320. doi: 10.1186/s12864-015-1517-1 (PMC4407392; doi:10.1186/s12864-015-1517-1)
Supplement: Additional file 2: Figure S2. — Sequence divergence between Cryptosporidium hominis IaA28R4 (specimen 30976) and IaA25R3 (isolate TU502) subtypes by chromosome. The number of the SNPs in a sliding window of 2,000 bp with 200 bp steps across each of the eight chromosomes is shown. [file 12864_2015_1517_MOESM2_ESM.pptx]

## Slide 1
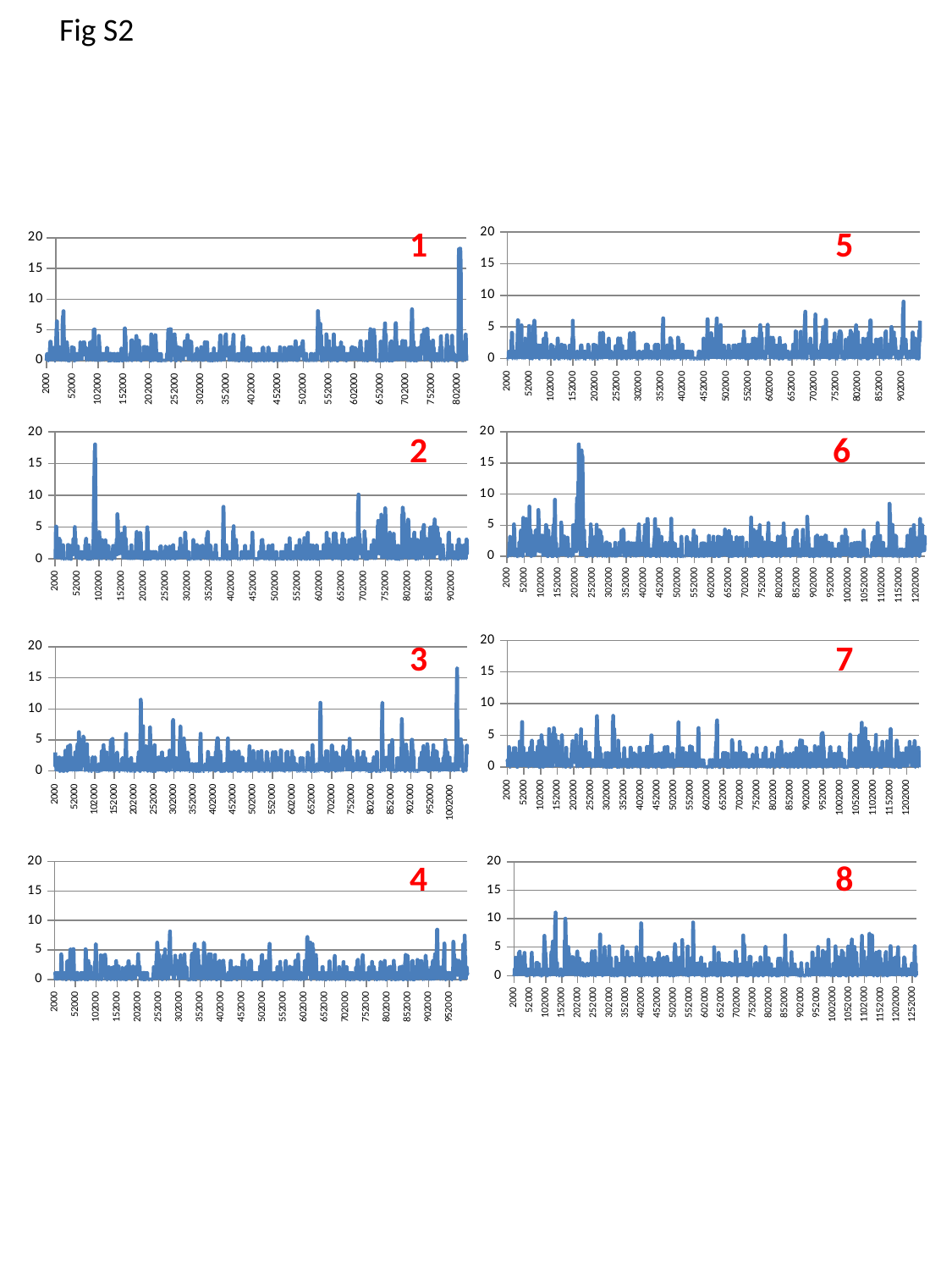

Fig S2
### Chart
| Category | |
|---|---|
### Chart
| Category | |
|---|---|1
5
2
6
### Chart
| Category | |
|---|---|
### Chart
| Category | |
|---|---|
### Chart
| Category | |
|---|---|
### Chart
| Category | |
|---|---|3
7
### Chart
| Category | |
|---|---|
### Chart
| Category | |
|---|---|4
8
