# Supplementary material for: Comparative genomic analysis reveals occurrence of genetic recombination in virulent Cryptosporidium hominis subtypes and telomeric gene duplications in Cryptosporidium parvum
Source: BMC Genomics. 2015 Apr 18;16(1):320. doi: 10.1186/s12864-015-1517-1 (PMC4407392; doi:10.1186/s12864-015-1517-1)
Supplement: Additional file 3: Figure S3. — Polymorphic nucleotide sequences in mid part of the Chro.60016 (ortholog of cgd6_60, coding for a protease) gene in Cryptosporidium hominis specimens. Dots denote sequence identity to the reference TU502 sequence. Specimen 37999 also has different sequence at the 3′ end of the gene (not shown). [file 12864_2015_1517_MOESM3_ESM.pptx]

## Slide 1
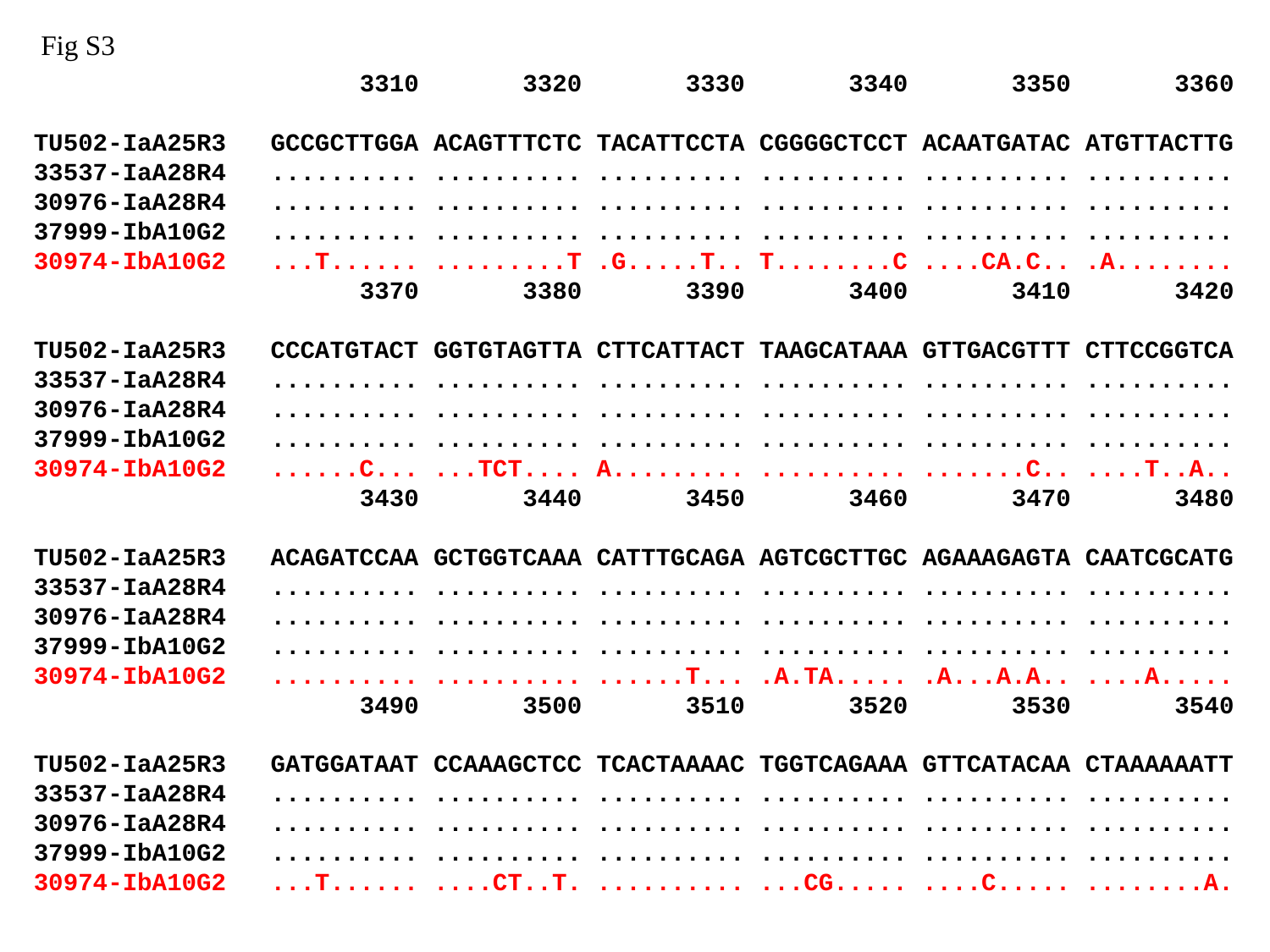

Fig S3
 3310 3320 3330 3340 3350 3360
TU502-IaA25R3 GCCGCTTGGA ACAGTTTCTC TACATTCCTA CGGGGCTCCT ACAATGATAC ATGTTACTTG
33537-IaA28R4 .......... .......... .......... .......... .......... ..........
30976-IaA28R4 .......... .......... .......... .......... .......... ..........
37999-IbA10G2 .......... .......... .......... .......... .......... ..........
30974-IbA10G2 ...T...... .........T .G.....T.. T........C ....CA.C.. .A........
 3370 3380 3390 3400 3410 3420
TU502-IaA25R3 CCCATGTACT GGTGTAGTTA CTTCATTACT TAAGCATAAA GTTGACGTTT CTTCCGGTCA
33537-IaA28R4 .......... .......... .......... .......... .......... ..........
30976-IaA28R4 .......... .......... .......... .......... .......... ..........
37999-IbA10G2 .......... .......... .......... .......... .......... ..........
30974-IbA10G2 ......C... ...TCT.... A......... .......... .......C.. ....T..A..
 3430 3440 3450 3460 3470 3480
TU502-IaA25R3 ACAGATCCAA GCTGGTCAAA CATTTGCAGA AGTCGCTTGC AGAAAGAGTA CAATCGCATG
33537-IaA28R4 .......... .......... .......... .......... .......... ..........
30976-IaA28R4 .......... .......... .......... .......... .......... ..........
37999-IbA10G2 .......... .......... .......... .......... .......... ..........
30974-IbA10G2 .......... .......... ......T... .A.TA..... .A...A.A.. ....A.....
 3490 3500 3510 3520 3530 3540
TU502-IaA25R3 GATGGATAAT CCAAAGCTCC TCACTAAAAC TGGTCAGAAA GTTCATACAA CTAAAAAATT
33537-IaA28R4 .......... .......... .......... .......... .......... ..........
30976-IaA28R4 .......... .......... .......... .......... .......... ..........
37999-IbA10G2 .......... .......... .......... .......... .......... ..........
30974-IbA10G2 ...T...... ....CT..T. .......... ...CG..... ....C..... ........A.
