# Supplementary material for: Comparative genomic analysis reveals occurrence of genetic recombination in virulent Cryptosporidium hominis subtypes and telomeric gene duplications in Cryptosporidium parvum
Source: BMC Genomics. 2015 Apr 18;16(1):320. doi: 10.1186/s12864-015-1517-1 (PMC4407392; doi:10.1186/s12864-015-1517-1)
Supplement: Additional file 4: Figure S4. — Lack of variation in sequence diversity in the trinucleotide repeat region in the gp60 gene of specimen 30974 of the Cryptosporidium hominis IbA10G2 subtype. Of 205 reads from 454 sequencing that mapped to gp60, 78 had complete sequence of the trinucleotide repeats, with no variation in repeat numbers. Dots denote sequence identity to the reference sequence, whereas dashes denote deletions of nucleotides. [file 12864_2015_1517_MOESM4_ESM.pptx]

## Slide 1
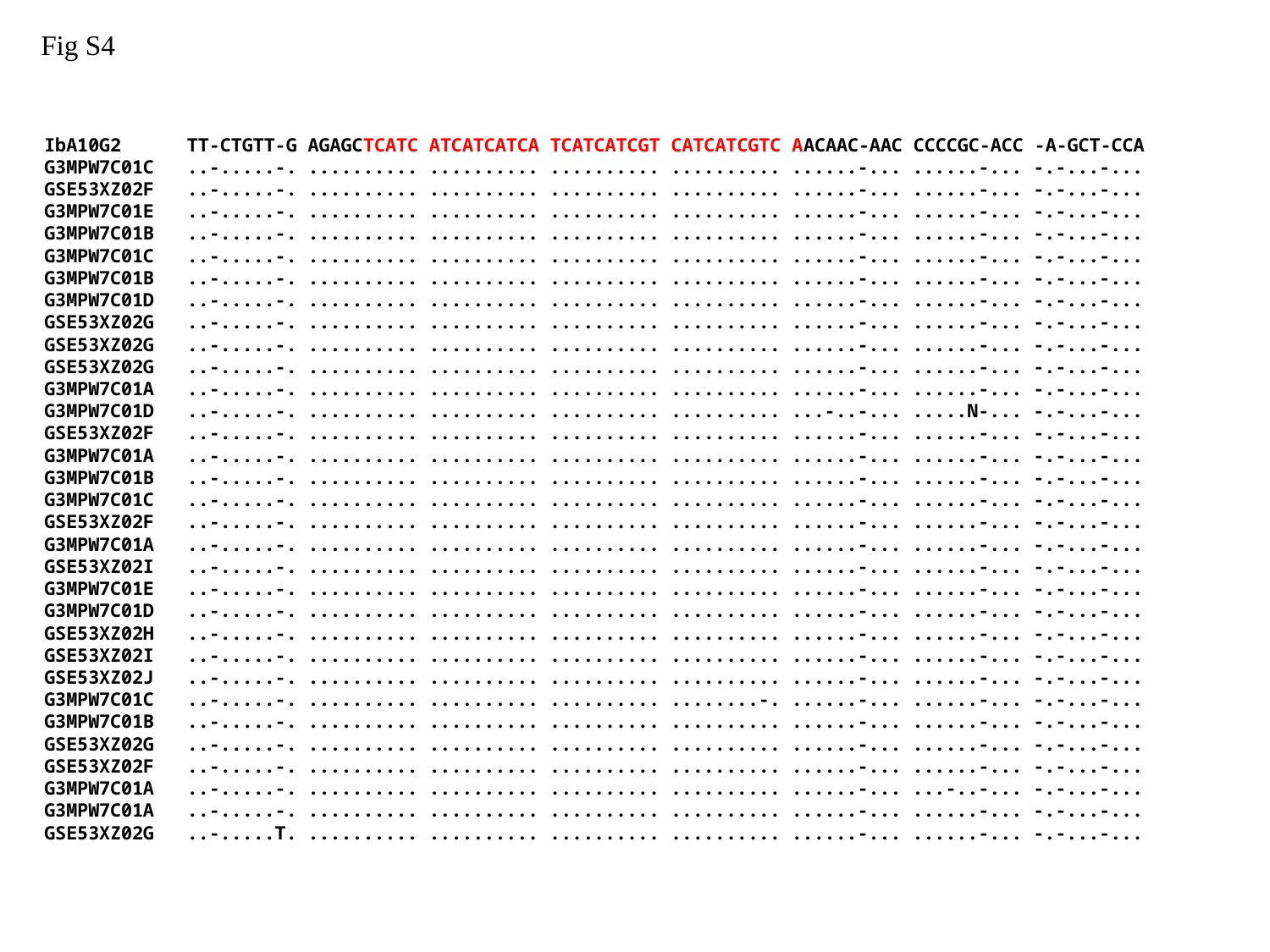

Fig S4
IbA10G2 TT-CTGTT-G AGAGCTCATC ATCATCATCA TCATCATCGT CATCATCGTC AACAAC-AAC CCCCGC-ACC -A-GCT-CCA
G3MPW7C01C ..-.....-. .......... .......... .......... .......... ......-... ......-... -.-...-...
GSE53XZ02F ..-.....-. .......... .......... .......... .......... ......-... ......-... -.-...-...
G3MPW7C01E ..-.....-. .......... .......... .......... .......... ......-... ......-... -.-...-...
G3MPW7C01B ..-.....-. .......... .......... .......... .......... ......-... ......-... -.-...-...
G3MPW7C01C ..-.....-. .......... .......... .......... .......... ......-... ......-... -.-...-...
G3MPW7C01B ..-.....-. .......... .......... .......... .......... ......-... ......-... -.-...-...
G3MPW7C01D ..-.....-. .......... .......... .......... .......... ......-... ......-... -.-...-...
GSE53XZ02G ..-.....-. .......... .......... .......... .......... ......-... ......-... -.-...-...
GSE53XZ02G ..-.....-. .......... .......... .......... .......... ......-... ......-... -.-...-...
GSE53XZ02G ..-.....-. .......... .......... .......... .......... ......-... ......-... -.-...-...
G3MPW7C01A ..-.....-. .......... .......... .......... .......... ......-... ......-... -.-...-...
G3MPW7C01D ..-.....-. .......... .......... .......... .......... ...-..-... .....N-... -.-...-...
GSE53XZ02F ..-.....-. .......... .......... .......... .......... ......-... ......-... -.-...-...
G3MPW7C01A ..-.....-. .......... .......... .......... .......... ......-... ......-... -.-...-...
G3MPW7C01B ..-.....-. .......... .......... .......... .......... ......-... ......-... -.-...-...
G3MPW7C01C ..-.....-. .......... .......... .......... .......... ......-... ......-... -.-...-...
GSE53XZ02F ..-.....-. .......... .......... .......... .......... ......-... ......-... -.-...-...
G3MPW7C01A ..-.....-. .......... .......... .......... .......... ......-... ......-... -.-...-...
GSE53XZ02I ..-.....-. .......... .......... .......... .......... ......-... ......-... -.-...-...
G3MPW7C01E ..-.....-. .......... .......... .......... .......... ......-... ......-... -.-...-...
G3MPW7C01D ..-.....-. .......... .......... .......... .......... ......-... ......-... -.-...-...
GSE53XZ02H ..-.....-. .......... .......... .......... .......... ......-... ......-... -.-...-...
GSE53XZ02I ..-.....-. .......... .......... .......... .......... ......-... ......-... -.-...-...
GSE53XZ02J ..-.....-. .......... .......... .......... .......... ......-... ......-... -.-...-...
G3MPW7C01C ..-.....-. .......... .......... .......... ........-. ......-... ......-... -.-...-...
G3MPW7C01B ..-.....-. .......... .......... .......... .......... ......-... ......-... -.-...-...
GSE53XZ02G ..-.....-. .......... .......... .......... .......... ......-... ......-... -.-...-...
GSE53XZ02F ..-.....-. .......... .......... .......... .......... ......-... ......-... -.-...-...
G3MPW7C01A ..-.....-. .......... .......... .......... .......... ......-... ...-..-... -.-...-...
G3MPW7C01A ..-.....-. .......... .......... .......... .......... ......-... ......-... -.-...-...
GSE53XZ02G ..-.....T. .......... .......... .......... .......... ......-... ......-... -.-...-...
Of 205 reads mapped to gp60, 78 had complete sequence of the trinucleotide repeats, with no variation in repeat numbers.
