# Supplementary material for: Comparative genomic analysis reveals occurrence of genetic recombination in virulent Cryptosporidium hominis subtypes and telomeric gene duplications in Cryptosporidium parvum
Source: BMC Genomics. 2015 Apr 18;16(1):320. doi: 10.1186/s12864-015-1517-1 (PMC4407392; doi:10.1186/s12864-015-1517-1)
Supplement: Additional file 5: Figure S5. — Lack of variation in sequence diversity in the trinucleotide repeat region in the gp60 gene of specimen 33537 of the Cryptosporidium hominis IaA28R4 subtype. Of 310 reads from 454 sequencing that mapped to gp60, 59 had complete sequence of the trinucleotide repeats, with no variation in repeat numbers. Dots denote sequence identity to the reference sequence, whereas dashes denote deletions of nucleotides. [file 12864_2015_1517_MOESM5_ESM.pptx]

## Slide 1
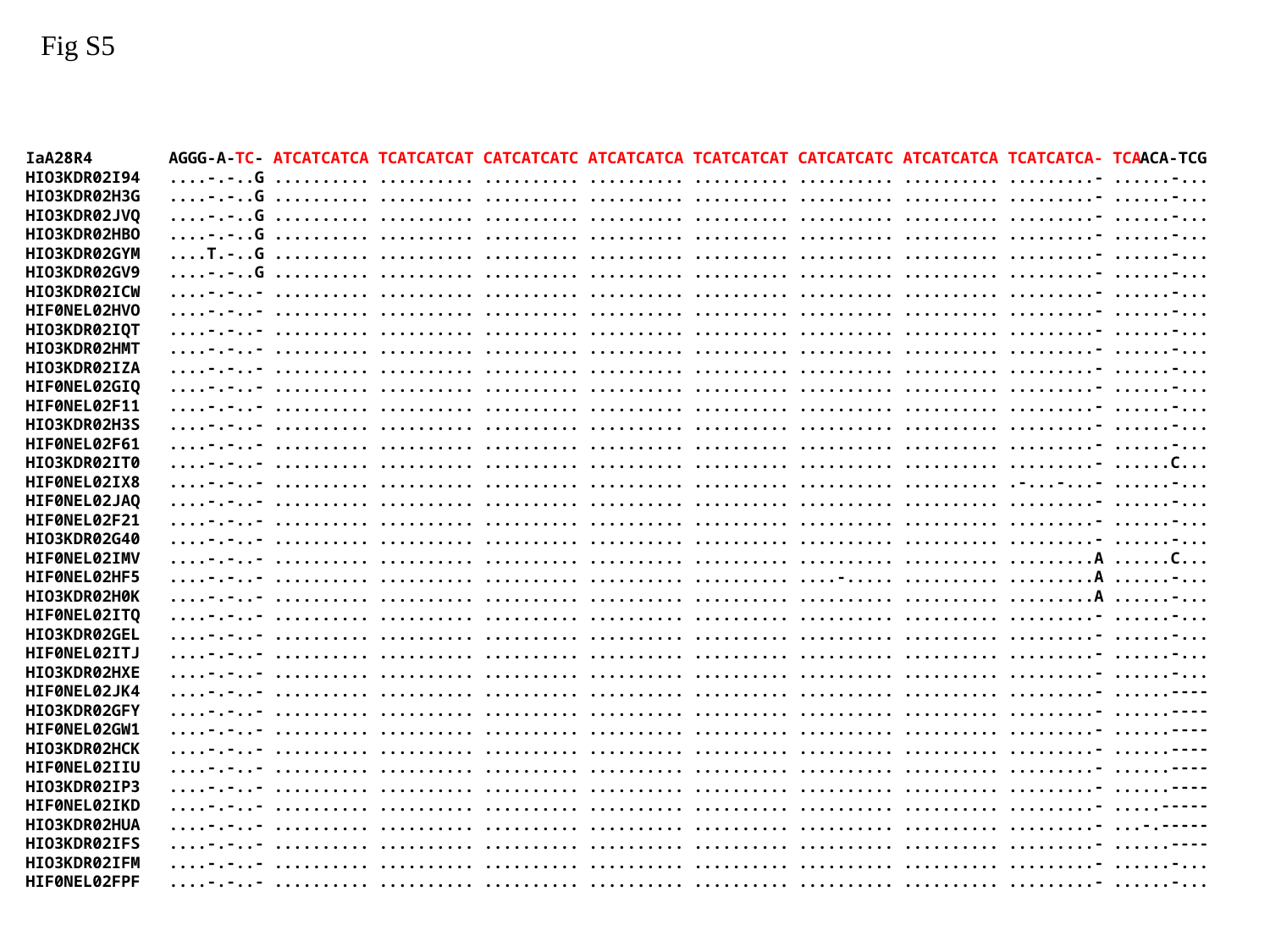

Fig S5
IaA28R4 AGGG-A-TC- ATCATCATCA TCATCATCAT CATCATCATC ATCATCATCA TCATCATCAT CATCATCATC ATCATCATCA TCATCATCA- TCAACA-TCG
HIO3KDR02I94 ....-.-..G .......... .......... .......... .......... .......... .......... .......... .........- ......-...
HIO3KDR02H3G ....-.-..G .......... .......... .......... .......... .......... .......... .......... .........- ......-...
HIO3KDR02JVQ ....-.-..G .......... .......... .......... .......... .......... .......... .......... .........- ......-...
HIO3KDR02HBO ....-.-..G .......... .......... .......... .......... .......... .......... .......... .........- ......-...
HIO3KDR02GYM ....T.-..G .......... .......... .......... .......... .......... .......... .......... .........- ......-...
HIO3KDR02GV9 ....-.-..G .......... .......... .......... .......... .......... .......... .......... .........- ......-...
HIO3KDR02ICW ....-.-..- .......... .......... .......... .......... .......... .......... .......... .........- ......-...
HIF0NEL02HVO ....-.-..- .......... .......... .......... .......... .......... .......... .......... .........- ......-...
HIO3KDR02IQT ....-.-..- .......... .......... .......... .......... .......... .......... .......... .........- ......-...
HIO3KDR02HMT ....-.-..- .......... .......... .......... .......... .......... .......... .......... .........- ......-...
HIO3KDR02IZA ....-.-..- .......... .......... .......... .......... .......... .......... .......... .........- ......-...
HIF0NEL02GIQ ....-.-..- .......... .......... .......... .......... .......... .......... .......... .........- ......-...
HIF0NEL02F11 ....-.-..- .......... .......... .......... .......... .......... .......... .......... .........- ......-...
HIO3KDR02H3S ....-.-..- .......... .......... .......... .......... .......... .......... .......... .........- ......-...
HIF0NEL02F61 ....-.-..- .......... .......... .......... .......... .......... .......... .......... .........- ......-...
HIO3KDR02IT0 ....-.-..- .......... .......... .......... .......... .......... .......... .......... .........- ......C...
HIF0NEL02IX8 ....-.-..- .......... .......... .......... .......... .......... .......... .......... .-...-...- ......-...
HIF0NEL02JAQ ....-.-..- .......... .......... .......... .......... .......... .......... .......... .........- ......-...
HIF0NEL02F21 ....-.-..- .......... .......... .......... .......... .......... .......... .......... .........- ......-...
HIO3KDR02G40 ....-.-..- .......... .......... .......... .......... .......... .......... .......... .........- ......-...
HIF0NEL02IMV ....-.-..- .......... .......... .......... .......... .......... .......... .......... .........A ......C...
HIF0NEL02HF5 ....-.-..- .......... .......... .......... .......... .......... ....-..... .......... .........A ......-...
HIO3KDR02H0K ....-.-..- .......... .......... .......... .......... .......... .......... .......... .........A ......-...
HIF0NEL02ITQ ....-.-..- .......... .......... .......... .......... .......... .......... .......... .........- ......-...
HIO3KDR02GEL ....-.-..- .......... .......... .......... .......... .......... .......... .......... .........- ......-...
HIF0NEL02ITJ ....-.-..- .......... .......... .......... .......... .......... .......... .......... .........- ......-...
HIO3KDR02HXE ....-.-..- .......... .......... .......... .......... .......... .......... .......... .........- ......-...
HIF0NEL02JK4 ....-.-..- .......... .......... .......... .......... .......... .......... .......... .........- ......----
HIO3KDR02GFY ....-.-..- .......... .......... .......... .......... .......... .......... .......... .........- ......----
HIF0NEL02GW1 ....-.-..- .......... .......... .......... .......... .......... .......... .......... .........- ......----
HIO3KDR02HCK ....-.-..- .......... .......... .......... .......... .......... .......... .......... .........- ......----
HIF0NEL02IIU ....-.-..- .......... .......... .......... .......... .......... .......... .......... .........- ......----
HIO3KDR02IP3 ....-.-..- .......... .......... .......... .......... .......... .......... .......... .........- ......----
HIF0NEL02IKD ....-.-..- .......... .......... .......... .......... .......... .......... .......... .........- .....-----
HIO3KDR02HUA ....-.-..- .......... .......... .......... .......... .......... .......... .......... .........- ...-.-----
HIO3KDR02IFS ....-.-..- .......... .......... .......... .......... .......... .......... .......... .........- ......----
HIO3KDR02IFM ....-.-..- .......... .......... .......... .......... .......... .......... .......... .........- ......-...
HIF0NEL02FPF ....-.-..- .......... .......... .......... .......... .......... .......... .......... .........- ......-...
