# Supplementary material for: Comparative genomic analysis reveals occurrence of genetic recombination in virulent Cryptosporidium hominis subtypes and telomeric gene duplications in Cryptosporidium parvum
Source: BMC Genomics. 2015 Apr 18;16(1):320. doi: 10.1186/s12864-015-1517-1 (PMC4407392; doi:10.1186/s12864-015-1517-1)
Supplement: Additional file 6: Table S1. — Primers used in the PCR verification of possible Cryptosporidium hominis-unique nucleotide sequences. [file 12864_2015_1517_MOESM6_ESM.docx]

**Table S1.** Primers used in the PCR verification of possible *Cryptosporidium hominis*-unique nucleotide sequences.

| Target | Gene | Primer | Sequence (5’ to 3’) | Size of expected PCR product (bp) |
| --- | --- | --- | --- | --- |
| AAEL01000413 (5’ end) | Chro.50011 | F1 | TAG GGT GAC AAC TTA ATG TGC C | ~571 |
|  |  | R1 | CCA TTA CTA CAC GAT CTC |  |
|  |  | F2 | GCC AAA TTC GAG GAG A |  |
|  |  | R2 | GTC AAA TGA ATG TCC GGT T |  |
| AAEL01000413 (mid) | Chro.50011 | F3 | TCA TTC AGA CTA CAG TAG CA | ~425 |
|  |  | R3 | GTC GTG TTA CTT GTC GTC T |  |
|  |  | F4 | GTT GTA AGT TCG AGA CTT GC |  |
|  |  | R4 | AGG TAA TGC TGG AAC TGG T |  |
| AAEL01000413 (3’ end) | Chro.50011 | F5 | TGT TCA GTA TCA ACA CCA GT | ~610 |
|  |  | R5 | TTG AAA TCC GCT GAT TGA CT |  |
|  |  | F6 | ATC AGC ATC ACT GAC GGT AC |  |
|  |  | R6 | CTG TGT ACA ACG AGT ACT A |  |
| AAEL01000728 | Unknown | F8 | aat caa cta aga ata ata tta gaa cc | ~413 |
|  |  | R8 | tga tgt gaa tcc aaa gtc tct tc |  |
| AAEL01000717 | Chro.00003 | F9 | ATT GGC GGA GAT GAA GCC GA | ~398 |
|  |  | R9 | CAG GTC CAG AAC GAT TAC ACG |  |
